# Resveratrol as a circadian clock modulator: mechanisms of action and therapeutic applications

**DOI:** 10.1007/s11033-023-08513-2

**Published:** 2023-05-25

**Authors:** Weronika Spaleniak, Muriel Cuendet

**Affiliations:** 1 School of Pharmaceutical Sciences, University of Geneva, Geneva, Switzerland; 2 Institute of Pharmaceutical Sciences of Western Switzerland, University of Geneva, Geneva, Switzerland

**Keywords:** Biological clock, Chrononutrition, Circadian rhythm, Nutraceuticals, Resveratrol, Sirtuin 1

## Abstract

In the past decades, resveratrol has gained increasing attention due to its versatile and beneficial properties. This natural polyphenol, commonly present in the human diet, has been shown to induce SIRT1 and to modulate the circadian rhythm at the cellular and organismal levels. The circadian clock is a system regulating behavior and function of the human body, thus playing a crucial role in health maintenance. It is primarily entrained by light-dark cycles; however, other factors such as feeding-fasting, oxygen and temperature cycles play a significant role in its regulation. Chronic circadian misalignment can lead to numerous pathologies, including metabolic disorders, age-related diseases or cancer. Therefore, the use of resveratrol may be a valuable preventive and/or therapeutic strategy for these pathologies. This review summarizes studies that evaluated the modulatory effect of resveratrol on circadian oscillators by focusing on the potential and limitations of resveratrol in biological clock-related disorders.

## Introduction

The circadian rhythm (*Circa diem*, from Latin, which means “about a day”) is an endogenous anticipatory system that is present in almost every light-sensitive organism on Earth. It enables adaptation to geophysical time changes associated with planet rotation around its axis by regulating the vast majority of the behavioral, physiological, and molecular processes [1, 2]. In mammals, this system constitutes the central clock located in the brain, and peripheral clocks found in almost every organ and tissue of the body. Light is the predominant environmental factor that synchronizes circadian rhythms in mammals on a daily basis [3]. When a light pulse falls on the retina in the eye, it is converted into electrochemical information transmitted to the hypothalamus. There, the central clock located in the suprachiasmatic nucleus (SCN) receives the signal and synchronizes the internal rhythms with the external light cycles. The central clock, also called the master pacemaker, synchronizes the web of peripheral oscillators through the nervous system, as well as humoral and non-humoral pathways [4, 5]. A study reported that the circadian system could control up to 80% of protein-coding genes in primates by orchestrating diverse biochemical processes [6]. In this regard, it is not surprising that any disturbances in the circadian clock could unbalance the body homeostasis. A desynchrony between the internal rhythm and external cues is called circadian misalignment and becomes a hallmark of our modern 24/7 lifestyle where the unlimited access to electricity and artificial light is common. A prolong misalignment may have disastrous consequences for human health such as obesity, diabetes, allergies, cancer, heart diseases, and mental disorders [7–11].

One example in which the circadian cycle plays a crucial role is metabolism. The circadian system adapts metabolic needs between the active and rest phases in mammals. As they eat mostly during their active phase, the metabolic processes related to food intake, such as insulin increase, nutrient uptake, and detoxification, are under circadian control and are activated when food is expected [12]. In addition, the composition of the meals and the resulting metabolic signals influence the circadian rhythm [13, 14]. This bidirectional interaction enables the flexibility needed to adapt the metabolism to the current body requirements and environmental conditions [15]. Several small molecules, such as caffeine, epigallocatechin gallate, and nobiletin, which are present in the human diet, were shown to modulate the circadian rhythm in vitro as well as in animal models [16]. For instance, nobiletin improved glucose tolerance and overall glucolipid metabolism through clock reprogramming in metabolic disordered hepatocytes [17], as well as in diabetic mice [18]. Moreover, it restored an attenuated circadian clock and improved insulin secretion as seen in isolated human type 2 diabetes pancreatic islets [19], as well as in mice [20]. An increasing body of evidence indicates that another dietary phytochemical, resveratrol, may also be a promising clock modulator [16].

Resveratrol (3,5,4’-trihydroxystilbene) has received a considerable amount of attention in the past decades due to a wide range of beneficial health effects [21]. This natural phytoalexin belongs to the stilbene family and is commonly present in the diet; it is mostly found in grape seeds and skin, red wine, peanuts, various types of berries, soy and cocoa [22–25]. Resveratrol can modulate numerous signaling molecules and pathways. As a result, there is growing evidence showing a positive impact of resveratrol on health issues, such as obesity, diabetes, cancer, cardiovascular diseases, liver diseases, Alzheimer’s and Parkinson’s diseases, but also viral and bacterial infections [21, 26–28]. The exploration of the link between resveratrol and the circadian rhythm shed a new light on possible applications of this unique phytochemical in clock-related diseases. However, despite a great deal of research on resveratrol, a summary of the findings on its role in clock regulation and chronobiology is still lacking.

This review presents studies related to the circadian clock modulation by resveratrol. A particular focus was set on the molecular mechanisms and therapeutic effects in related pathologies, as well as general limitations of resveratrol application in humans.

## Molecular mechanisms of circadian rhythm regulation and the role of resveratrol

**Fig. 1:**
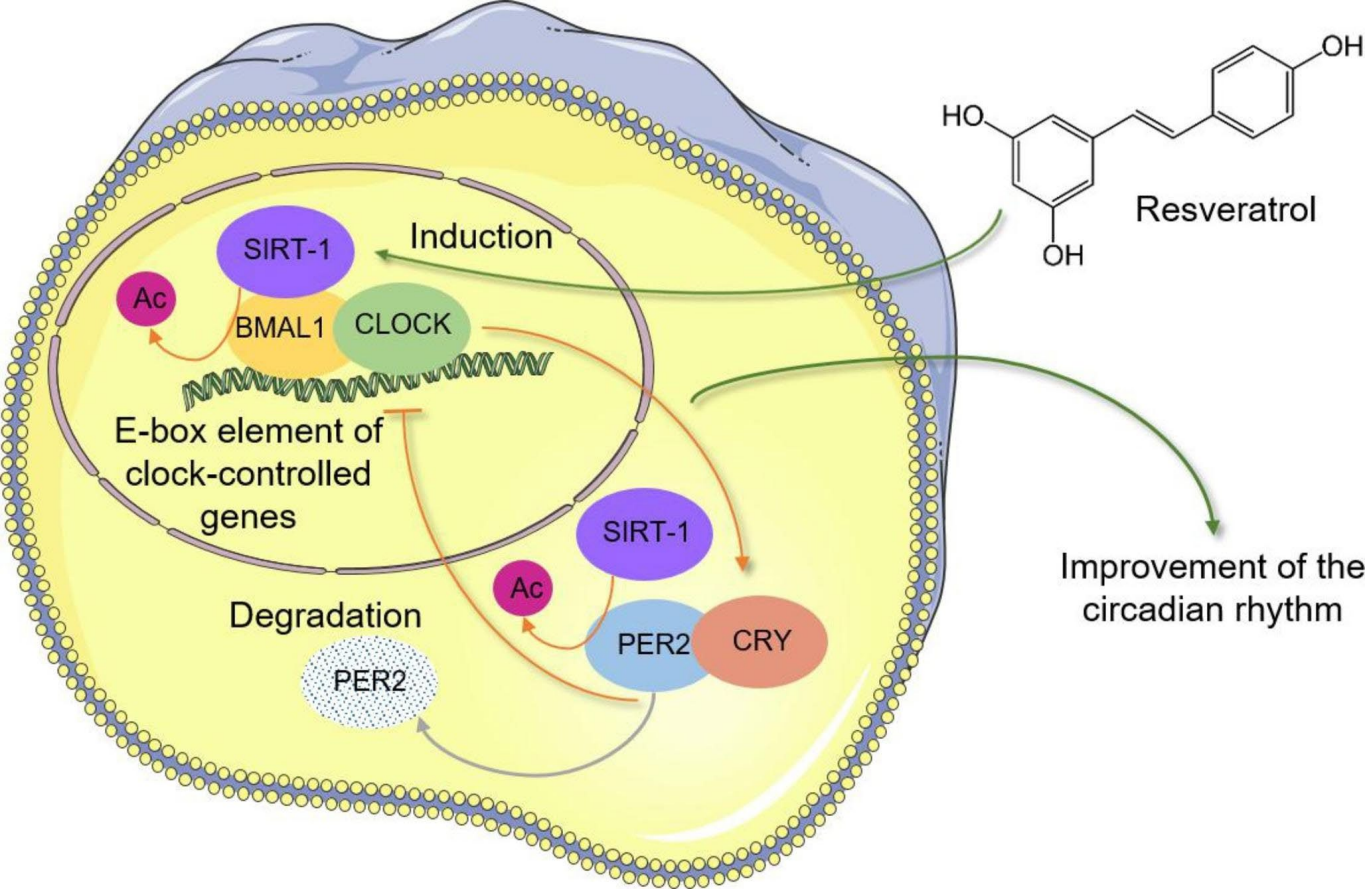
The Main TTFL of the circadian clock regulation and the effect of resveratrol BMAL1/CLOCK heterodimer binds to E-box element of target genes, such as *Per2* and *Cry*, which are negative clock components. Once expressed, they inhibit BMAL1 and CLOCK expression. Resveratrol induces SIRT1, which deacetylases BMAL1 resulting in the enhancement of its activity. SIRT1 was also shown to deacetylate PER2 leading to its degradation

The molecular regulation of the circadian clock is based on transcription-translation feedback loops (TTFL). The main loop is composed of the Brain and Muscle Arnt-like protein 1 (BMAL1) and the Circadian Locomotor Output Cycles Kaput (CLOCK) that are positive regulators. These transcription factors form a heterodimer and induce the transcription of genes containing the E/E′-box element in the promoter or enhancer regions. These are, among others, period (*PER*) and cryptochrome (*CRY*) genes that are the negative components of the main loop. When their level reaches a critical point, they form a protein complex that inhibits CLOCK-BMAL1 activity in a negative feedback loop. Consequently, PER and CRY levels decrease, and *CLOCK* and *BMAL1* expression can be restored, which closes the 24 h cycle (Fig. 1). The auxiliary loop of the core-clock machinery consists of the nuclear receptors reverse erythroblastosis viruses α and β (REV-ERBα/β), as well as the retinoid-related orphan receptors α, β and γ (RORα/β/γ). REV-ERBα/β and RORα/β/γ compete for binding to REV-ERB-ROR response element, which is also present in *BMAL1* promoter and enhancer regions. REV-ERBα/β has an inhibitory effect on BMAL1 transcription, while RORα/β/γ binding activates it [3].

SIRT1 was shown to be an important circadian regulator [29]. *SIRT1* codes for Sirtuin 1, the best studied member of the sirtuin protein family. It plays a role in metabolic and physiological processes through its ability to deacetylate histone and non-histone molecules. SIRT1 removes an acetyl group from the target protein using an NAD^+^-dependent mechanism [30]. NF-κB, p53 and peroxisome proliferator-activated receptor gamma-coactivator-1α (PGC-1α) are among the numerous targets of this protein. The BMAL1-CLOCK complex could induce *SIRT1* transcription through binding to two E-box elements of the *SIRT1* promoter region [31]. Circadian clock regulated SIRT1 mRNA and protein expression, as well as its activity. This was shown in a circadian misalignment model consisting of mice kept in constant darkness for 14 days. Animals had decreased BMAL1 and SIRT1 protein levels, lower SIRT1 activity, as well as increased acetylation of p53 and RelA/p65, which are two targets of SIRT1 [31]. Besides being regulated by the clock, SIRT1 could also affect the circadian regulation itself by being a positive regulator of the circadian rhythm. Studies showed that *Bmal1* and *Sirt1* mRNA expressions were highly correlated, therefore suggesting mutual positive regulation [32]. SIRT1 influenced BMAL1 expression through PGC-1α [33] and enhanced BMAL1 activity by deacetylation. It resulted in an improvement of the amplitude of the central circadian clock [34] (Fig. 1). SIRT1 was shown to bind to the BMAL1-CLOCK heterodimer [35–37] and the protein complexes were found in the nuclei – probably due to SIRT1 nuclear localization sequence. The PAS-B domain of BMAL1 was identified as a possible region to interact with SIRT1 [37]. SIRT1 also improved the circadian clock by inhibiting the transcription of the negative clock component *Per2* (Fig. 1) [36]. SIRT1 deacetylated PER2, which resulted in its degradation [33, 35]. On the contrary, some studies suggested that SIRT1 was a negative regulator of the clock. Deacetylation of BMAL1 by SIRT1 resulted in destabilization of the protein and disturbed the circadian rhythm [38]. Further investigations are therefore needed to fully understand the role of SIRT1 in body clock regulations.

Resveratrol was shown to be an activator of *SIRT1* expression [39, 40] and it may consequently impact the circadian rhythm [29, 31, 35, 38]. The first evidence appeared in 2008 when Oike and Kobori observed that treatment with resveratrol modulated circadian rhythm-related genes in Rat1 fibroblast cells [41]. A 100 µM dose significantly increased the expression of *Bmal1, Per1* and *Per2*. Moreover, the mechanism of circadian regulation by the compound differed between serum shock, forskolin or glucose treatment, which are well known entrainers of the circadian rhythm. Treatment with resveratrol showed similar inhibitory effect on *Per2* transcription than SIRT1, suggesting an impact on circadian rhythm through SIRT1 [36]. Nonetheless, resveratrol (100 µM) was not confirmed to have a direct effect on SIRT1 interaction with the clock proteins [37]. In the same study, resveratrol reduced the transcriptional activity of *Ebox* and *Per1* promoters. It also reduced CLOCK/BMAL1-mediated *Per1* promoter activity when SIRT1 was co-expressed in the cells. The authors concluded that resveratrol induced SIRT1, which acted as a negative clock regulator due to deacetylation and repression of *Per1* activity [37]. This stands in opposition to most other studies.

## Resveratrol as a modulator of the circadian rhythm in various diseases

### Metabolic disorders

Circadian rhythm misalignment can lead to various metabolic disorders such as dyslipidemia, hyperglycemia, insulin resistance, obesity and diabetes [7, 8]. Resveratrol showed a wide range of positive effects in metabolic disorders mainly due to SIRT1 up-regulation [26]. The investigations on the link between resveratrol, circadian rhythm and metabolic disorders seems to be a valid approach to develop therapeutic strategies against them.

#### Lipid and glucose metabolism

A recent in vitro study investigated the effect of resveratrol on the metabolic state and molecular clock in AML-12 mouse hepatocytes [42]. Cells treated with 50 µM resveratrol for 6 h exhibited a decrease in the ratio between phosphorylated proteins and total level of several metabolic markers, such as protein phosphatase 2 (PP2A), AKT, FOXO1, mTOR, and AMPK. These changes indicated an inhibition of AMPK and an induction of the PP2A-FOXO1-PEPCK pathway, which is suggesting gluconeogenesis induction and a fasting state in the cells. Resveratrol also induced a decrease in the ratio between phosphorylated BMAL1 and BMAL1. Moreover, compound treatment caused phase advance and reduced the amplitude of *BMAL1*, *SREBP1* and *PGC1Α* mRNA oscillations. On the other hand, the *SIRT1* gene phase was delayed and its oscillation amplitude decreased. Together, this study suggested that resveratrol altered the metabolism and circadian rhythm of hepatocytes by mimicking fasting state activation.

**Fig. 2:**
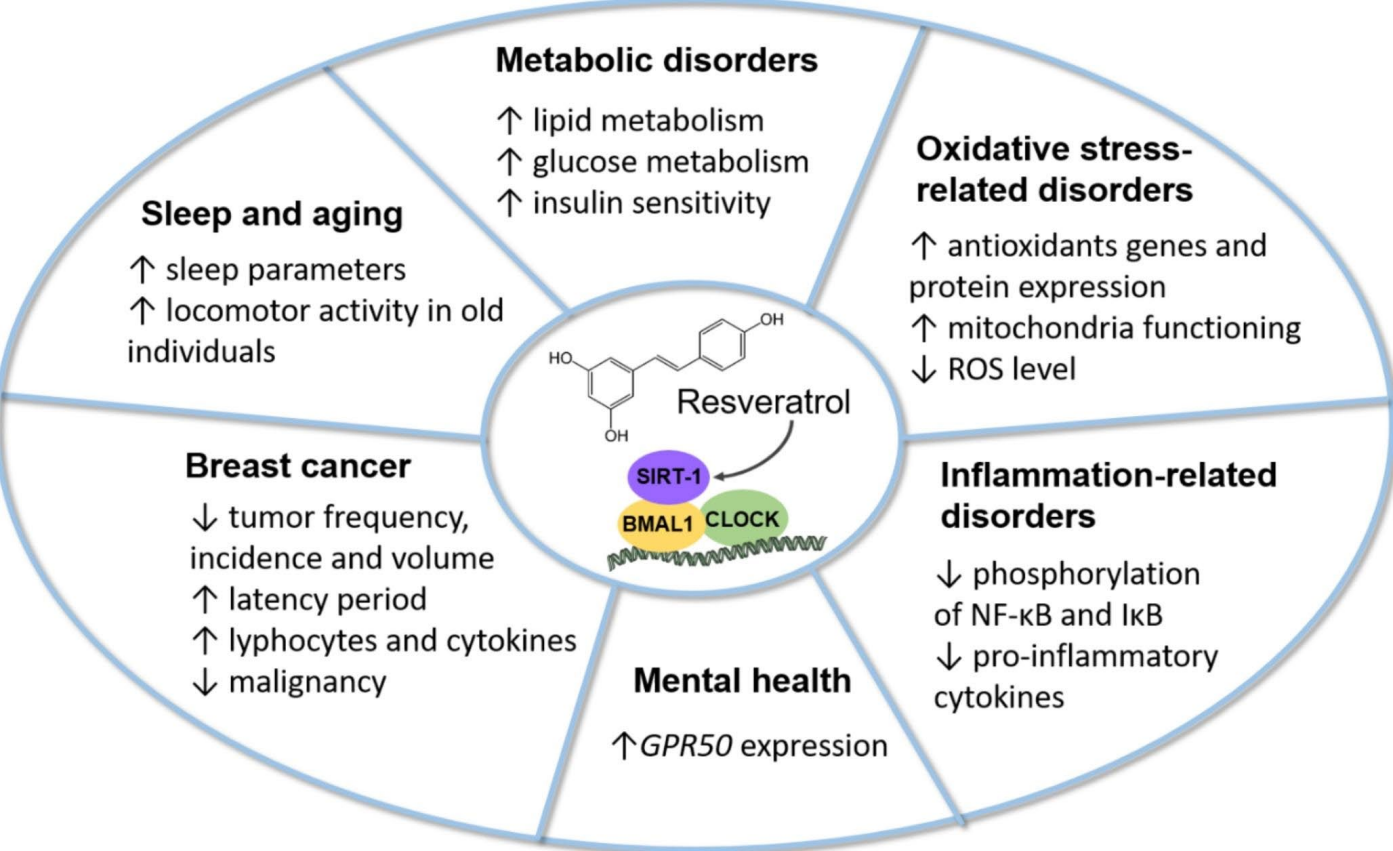
Beneficial effects of resveratrol through interaction with the circadian clock in various diseases. ↑ indicates an increase or improvement and ↓ indicates a decrease

In another study, free fatty acids (FFA) were shown to cause circadian misalignment in hepatic HepG2 cells [43]. FFA down-regulated and attenuated oscillation amplitude of circadian clock-related gene expression, such as *BMAL1*, *CLOCK*, *CRY1*, *PER1*, *PER2* and *REVERB-Α.* It also caused phase shift of *BMAL1* and *CLOCK*. Pretreatment with resveratrol (100 µM) for 6 h restored these changes. Decreased protein expression levels (BMAL1, CLOCK and SIRT1) induced by FFA and their phase shift were also partially reversed by resveratrol. Besides, FFA reduced phosphorylation of acetyl-CoA carboxylase, AMPK, AKT and insulin receptor substrate 1 (IRS-1). It also up-regulated the expression of lipogenesis proteins, inhibited GSK-3 activation, as well as led to the accumulation of triglycerides (TG) and total cholesterol. Resveratrol partially or totally prevented all these changes and therefore protected the cells from the negative effects of FFA treatment on lipid and glucose metabolism [43] (Fig. 2). Importantly, all of these resveratrol effects were proven to be BMAL1-dependent using knockdown experiments.

Sun et al. showed that a high fat diet administered to C57BL/6 mice altered gene expression and rhythmicity of *Bmal1*, *Clock*, *Per2, Pparα, Sirt1*, and lipogenesis genes such as *Acc1, Fas* and *Srebp-1c* in the liver [44]. The administration of high fat diet containing resveratrol (0.1% (w/w)) restored circadian oscillation of most studied genes. SIRT1 protein level was down-regulated under high fat diet conditions, which confirmed its role in metabolism disorders and circadian rhythm disruption. Resveratrol increased the rhythmicity of *Sirt1* leading to the restoration of circadian expression of lipogenesis and clock-related genes such as *Acc1*, *Fas*, *Pparα* and *Srebp-1c*. The high fat diet also contributed to an impaired rhythmicity of plasma lipids in mice, such as total cholesterol, TG, LDL, and plasma HDL, as well as increased plasma leptin and insulin levels. Resveratrol restored these changes and improved the respiratory exchange ratio and heat production. Moreover, resveratrol supplementation significantly reduced body weight and improved the fasting blood glucose compared to animals fed only with a high fat diet (Fig. 2).

Another study evaluating the effect of resveratrol in rats fed with a high fat diet showed that resveratrol administered in food supplemented with the compound (30 mg/kg/day for 6 weeks) could not improve the dysregulation of clock and adipogenic gene expression that occurred in epididymal adipose tissue and in the liver, except for a down-regulation of *Rev-Erbα* [45]. However, the activity of the fatty acid synthase was reduced and body weight in resveratrol-treated rats was significantly lower, suggesting that resveratrol could prevent adipogenesis and lipogenesis in high fat diet conditions via *Rev-Erbα*. Taken together, resveratrol could restore the negative effects of a fat diet through circadian rhythm improvement and therefore protect against metabolic-related diseases, such as dyslipidemia, dysglycemia and obesity (Fig. 2).

#### Insulin sensitivity

Insulin sensitivity is also closely related to circadian rhythm disturbances. mRNA and protein levels of CLOCK and BMAL1 were down-regulated in insulin-resistant primary mouse hepatocytes [31] and C2C12 myotubes [46], while ectopic expression of CLOCK and BMAL1 proteins increased insulin sensitivity. Knockdown experiments confirmed that *BMAL1*, *CLOCK* and *SIRT1* played important roles in insulin signaling and circadian variations in insulin sensitivity. SIRT1 ectopic expression significantly reduced insulin resistance in hepatocytes and in muscle cells in which CLOCK and BMAL1 were knocked down.

Similar observations on insulin resistance were made in diabetic mice [31, 46]. CLOCK and BMAL1 mRNA and protein levels were decreased in the liver and skeletal muscle of insulin resistant mice, and the ectopic expression of CLOCK and BMAL1 improved hepatic insulin sensitivity [31]. It was also shown that CLOCK, BMAL1 and SIRT1 were regulators of insulin sensitivity in vivo. In a circadian misalignment model (mice kept in the dark for 14 days) in which lower SIRT1 activity was observed, SIRT1 overexpression led to improved insulin sensitivity [31]. Treatment with resveratrol (25 mg/kg/day for 14 days in normal conditions) followed by 22 days in the dark prevented the decrease in SIRT1 activity in the liver and skeletal muscle of mice when in the dark [31, 46]. It also improved glucose and insulin intolerance. The treatment reverted impaired insulin signaling manifested by a decrease in phosphorylation of insulin receptor, Akt and GSK3β in both cell types. Resveratrol at a lower dose (2.5 mg/kg/day) for 5 days (followed by 22 days in the dark) also showed positive outcomes in terms of insulin tolerance and cell signaling in the liver [31]. These studies showed that resveratrol had beneficial effects on insulin resistance by modulating clock components (Fig. 2).

### Oxidative stress-related disorders

Reactive oxygen species (ROS) imbalance causes protein, lipid and DNA damages that can lead to cell dysfunction and even carcinogenesis [47]. Oxidative stress response is clock dependent, as many of the antioxidant enzymes display circadian variations [48–50] and BMAL1 plays an important role in their regulation [51]. Moreover, BMAL1 maintains redox balance [52], and mitochondrial function [53, 54]. In physiological conditions, cellular defense systems are able to maintain redox homeostasis. In primary mice hepatocytes, acrylamide, a toxic compound causing redox imbalance and oxidative damage, disturbed the clock function by decreasing oscillatory amplitudes of *Bmal1* and *Clock*, leading to their phase shift [55]. It also enhanced *Cry1, Cry2* and *Per1* amplitude, causing phase shift of *Per1*, and decreased mRNA level of *PGC-1α* and *Sirt1.* Moreover, it reduced protein levels of BMAL1, CLOCK, CRY1 and SIRT1. Resveratrol pretreatment (50 µM) for 4 h prevented these effects. In HepG2 cells, acrylamide led to a decrease in nuclear factor erythroid 2-related factor 2 (Nrf2) expression and its downstream antioxidant protein, NAD(P)H quinone dehydrogenase 1 (NQO1), which was reversed by resveratrol. The polyphenol also prevented mitochondrial dysfunction in HepG2 cells manifested as calcium and ATP imbalance, loss of mitochondrial membrane potential and decrease in mitochondrial complex protein levels (Fig. 2). Importantly, the effects on the Nrf2 pathway and mitochondrial dysfunction were noticed to be BMAL1-dependent, which was shown in a knockdown model. Resveratrol also reduced cytotoxicity and prevented morphological changes caused by acrylamide [55]. Moreover, resveratrol (100 µM) partially prevented the increase in intracellular ROS as well as the decrease in mitochondrial membrane potential in HepG2 cells treated by FFA [43]. Decrease in catalase (CAT) activity and expression inhibition of respiratory chain complexes I and III were partially restored. Overall, resveratrol improved the hepatic lipid metabolism and mitochondria dysfunction through restoration of the antioxidant response and elimination of excessive ROS in a clock dependent manner (Fig. 2).

Dysregulation of the circadian rhythm and the aberrant antioxidant response were also observed in Parkinson’s disease models using SH-SY5Y cells and rats treated with 6-hydroxydopamine [56]. In the cells, a decrease was observed in mRNA levels of *Bmal1, Clock, Per2*, and *Rorα*, as well as in the level of antioxidant genes, namely *Cat*, glutathione peroxidase (*Gpx*), glutathione S transferase (*Gst*) and superoxide dismutase (*Sod*). In rats, the same was observed except for an increase in *Rorα* and the alteration of *Sod* circadian rhythm with a shift of its peak by 12 h. Dysregulation of the circadian rhythm also occurred at the protein level. 6-Hydroxydopamine decreased BMAL1, CRY1 and SIRT1 protein levels in both models through most of the 24 h cycle. Simultaneously, the level of acetylated BMAL1 increased, indicating that SIRT1 deacetylated BMAL1. It was also noticed that the interaction between BMAL1 and CRY proteins increased. In order to further investigate the role of resveratrol in Parkinson’s disease and antioxidant response, cells were pretreated with 50 µM of resveratrol for 12 h. Resveratrol partially prevented the effects of 6-hydroxydopamine with an increase in *Cry1*, *Per2* and *Cat* mRNA levels and a decrease in acetylated BMAL1 level. Moreover, resveratrol reduced the BMAL1 and CRY1 interaction, suggesting it may improve the BMAL1-CLOCK interaction and expression of their target genes.

BMAL1 has been recently identified as a renal function regulator and it plays an important role in mitochondrial protection in ischemia reperfusion injury through the SIRT1/PGC-1α axis [57]. *BMAL1* knockdown in human kidney HK-2 cells decreased SIRT1 level, as well as increased mitochondrial damage and apoptosis caused by hypoxia/reoxygenation. Resveratrol was able to partially restore mitochondrial biogenesis in *BMAL1* knockdown HK-2 cells cultured under hypoxia/reoxygenation conditions through increased SIRT1 activity. In rats receiving 25 mg/kg/day of resveratrol for 2 weeks before ischemia-reperfusion, kidney cell apoptosis was partially prevented and antioxidant protein levels, as well as SIRT1 and PGC-1α protein levels were increased in the kidneys compared to control animals. Resveratrol also prevented vacuolization of mitochondria and mitochondrial biogenic dysfunction. Taken together, resveratrol administration partially prevented cell damage in HK-2 cells under hypoxia/reoxygenation conditions and alleviated the negative consequences of ischemia-reperfusion in rat kidney through SIRT1 induction [57].

The circadian variation of the thiobarbituric acid reactive species (TBARS) level was shown in different rat organs [58]. The TBARS level in the heart, liver and kidney was higher in dark span compared to light span. Since rats are nocturnal animals, authors suggested that it could be related to oxidative burst after food intake or changes in the anti- and pro-oxidative activity ratio in the organs. To investigate the antioxidant properties of resveratrol, the compound was administrated intraperitoneally to rats at different concentrations (0.8, 2, and 5 mg/kg) in the middle of the dark and light spans. Four hours after administration, TBARS level was measured. Resveratrol decreased TBARS level in the heart during the dark span in a dose-dependent manner, but when administrated during the light span, the outcome was opposite – it led to a dose-dependent increase in TBARS. Similar trends were observed in rat liver and kidney.

Taken together, the antioxidant response was closely linked to circadian rhythm regulation. Resveratrol, as an antioxidant and SIRT1 inducer, may be valuable in related disorders, such as Parkinson’s disease or exposition to toxic compounds. However, the time of resveratrol administration seems to be crucial for achieving the desired antioxidant effect and has to be considered in in vivo studies.

### Inflammation-related diseases

NF-κB pathway activation and pro-inflammatory cytokine expression, which are the causes of several chronic inflammatory diseases, were shown to be partially regulated by CRY proteins [59]. Acrylamide induced an inflammatory response in HepG2 cells through NF-κB and IκB phosphorylation as well as the expression of the pro-inflammatory cytokines TNF-α, iNOS, and IL-6 [55]. Pretreatment with resveratrol (50 µM for 4 h) prevented these events (Fig. 2). However, when *Cry1* gene was silenced, the protective effect of resveratrol was not observed. This indicated that resveratrol anti-inflammatory properties was dependent on the circadian rhythm [55].

### Aging and sleep regulation

Sleep is regulated by the circadian rhythm, and it is an indicator of normal human brain function and health. The biological clock naturally changes with aging. This results in sleep behavior disturbances. Sleep time shifts to earlier hours and is characterized by frequent awakenings and shortening of slow wave sleep (SWS) [60]. Growth differentiation factor 11 (GDF11), a cytokine belonging to the TGFß family, was shown to decrease with age and its restoration may bring beneficial effects in age-related diseases [61, 62]. Given its effects on circadian rhythm regulations, resveratrol may modulate age-related genes [32]. When human lung fibroblasts at low passage number (20) were compared to cells at high passage number (60), a 4 h treatment with resveratrol (100 µM) led to an increase in *BMAL1*, *REV-ERBα* and *SIRT1* mRNA levels in cells with a low passage number while the levels of *GDF11*, *NRF2*, *PER1*, and *SIRT6* were decreased. In high passage number cells, the effects of resveratrol differed partially – *GDF11*, *PER1*, and *SIRT6* levels increased and *SIRT1* expression decreased. The increase in *GDF11* induced by resveratrol in old cells may suggest an anti-aging effect. At the protein level, resveratrol down-regulated glucocorticoid receptor α (GRα) expression and up-regulated BMAL1 and SIRT1 levels in low passage cells. In high passage fibroblasts, resveratrol did not show any effect on BMAL1 and SIRT1 levels. Based on these results and a calculated correlation between the genes and proteins, the authors suggested that resveratrol induced BMAL1 protein expression in young and old cells through SIRT6 down-regulation, which is contradictory to most of the studies indicating that this polyphenol rather acts through SIRT1 up-regulation.

To study the effect resveratrol has on sleep, non-human primates (mouse gray lemurs) were treated with 200 mg/kg/d resveratrol for 3 weeks in long-day conditions (light:dark 14:10) and electroencephalographic rhythms were analyzed [63]. Reduced SWS time (-33%) and paradoxical sleep (PS) (-95%) were observed. The active wake (AW) phase increased (+ 45%), mainly during the resting phase (Fig. 2). There were no changes in body weight or body temperature. The reduced need for sleep induced by resveratrol supplementation in lemurs may be explained by an improved metabolism. PS, SWS, and their ratio may be indicators of the body metabolic status [64]. As already mentioned, circadian rhythm and metabolism are strongly linked and therefore an improved metabolism may impact sleep regulation. Another explanation relies on the antioxidant properties of the compound. It has been suggested that sleep is needed to protect the body against the negative effects of ROS produced during metabolic processes [65]. Lower brain temperature and slower metabolism during rest allows for a more efficient enzyme renewal. Resveratrol improved redox homeostasis and consequently could lead to lower sleep need. In summary, results suggested that resveratrol could modulate sleep-wake cycles and as a consequence may influence the circadian rhythm and metabolism regulations.

The impact of resveratrol on the circadian rhythm in grey mouse lemurs was also investigated in relation to age [66]. Young and old lemurs, which are nocturnal animals, received 200 mg/kg of the compound daily. Subsequently locomotor activity and body temperature were measured to investigate changes in the biological clock of the animals. After 4 weeks of supplementation, the locomotor activity of old lemurs significantly increased and showed similar values to young individuals (Fig. 2). In both groups, the locomotor activity onset (the time between activity onset and the start of the dark phase) was reduced, suggesting a better adaptation to light and dark phase changes. The night body temperature did not change significantly over the 4 weeks. However, the day body temperature increased in both age groups, and was significantly higher in old lemurs compared to young ones during most of the course of the experiment. Lemurs naturally undergo daily torpor, during which the body temperature decreases and the metabolism slows down allowing them to save energy [67–69]. Daily hypothermia shortened in both age groups and the minimal temperature increased, suggesting an influence of resveratrol on energy metabolism. The difference in effect of resveratrol supplementation between young and aged animals may be due to the deregulation of the circadian clock in elderly individuals (reduction of the active phase and increase in the rest phase). Aged animals may therefore benefit more from the effect of resveratrol.

### Mental disorders

There is evidence that circadian clock disturbances contribute to a variety of psychiatric disorders [11]. Melatonin is a crucial player in circadian rhythm regulation. However, its action can be disturbed by G protein coupled receptor 50 (GPR50), which forms a heterodimer with the melatonin receptor MT1 and prevents melatonin binding [70]. A certain polymorphism of *GPR50* seems to be connected to an elevated risk of bipolar disorder, schizophrenia and major depression in women [71]. It has been observed that *Gpr50* is a target of SIRT1 [72] and treatment of HEK-293 cells with resveratrol (50 µM) for 48 h increased *SIRT1* and *GPR50* gene expressions, as well as SIRT1 protein expression. Phospholipase C is an enzyme necessary for the transduction of photoperiodic signals and is activated via melatonin receptors. To investigate the effect of melatonin on phospholipase C activity in brain cells, SH-SY5Y human neuroblastoma cells were differentiated into dopamine neuronal phenotypes and treated with melatonin. Phospholipase C activity was increased, indicating that the photoperiodic signal was successfully transduced via melatonin receptors. Resveratrol treatment suppressed this effect through SIRT1, which was confirmed in SIRT1 knockdown cells. This suggests that SIRT1 was involved in melatonin signaling, possibly due to GPR50 activation. In vivo, 4 weeks of a diet supplemented with resveratrol induced an up-regulation of *Gpr50* level in the brain of Sprague-Dawley rats (Fig. 2), but no effect was observed on SIRT1 gene or protein expression. These findings indicate the potential effect of resveratrol consumption on sleep-wake cycles and brain functioning, and therefore its possible benefit for mental disorder therapies.

### Cancer and chemoprevention

The circadian clock also regulates key processes involved in cancer development and progession including cell cycle, apoptosis, metabolic regulation and DNA damage response [9]. To date there are only few publications linking the circadian rhythm, carcinogenesis and resveratrol. Melatonin, besides being a crucial circadian regulator, has also shown to inhibit breast cancer growth, angiogenesis, cancer cell invasion, and telomerase activity [73]. Sprague-Dawley rats were given food supplemented with resveratrol (100 mg/kg) and water containing 20 mg/l of melatonin [74]. The treatment reduced the incidence of mammary tumors as well as the number of invasive tumors (Fig. 2). As melatonin production varies with a peak during the night, the authors decided to investigate if a night administration of resveratrol impacted ER-positive breast cancer formation [75]. Therefore, rats received resveratrol 4 h after the beginning of the dark phase, which is supposed to overlap with *Per2* and melatonin peaks. In the group treated with resveratrol, reduced body weight was noticed. Tumor frequency and incidence, as well as tumor volume were significantly reduced, and the latency period extended compared to animals treated only with a carcinogen. In addition, an increased number of lymphocytes and higher levels of cytokines in the serum (IL-1A, IL-1B and IL-2) were observed, which suggests that resveratrol stimulated the immune system. ROS level was elevated in leukocytes. Resveratrol also improved metabolic parameters that are typical of carcinogenesis, namely hypoproteinemia and elevated urea concentration in the blood. The treatment increased total protein amount and reduced urea level (Fig. 2). These results should encourage further exploration of the relationship between resveratrol anticancer properties and the circadian clock.

## Clinical studies

Despite strong in vitro and in vivo links between circadian clock and resveratrol, only one clinical trial refers to its chronobiology [76]. The study focused on pharmacokinetics and safety of orally administrated resveratrol in healthy humans. The compound was given in the form of capsules every 4 h for 48 h at different doses (25, 50, 100 and 150 mg). Resveratrol was well tolerated by the participants with rare to mild adverse effects. Nonetheless, the bioavailability and blood concentration remained low despite relatively high doses and short intervals between them. Interestingly, diurnal variations in terms of pharmacokinetics were observed – resveratrol concentrations in the blood reached the highest values after morning administration and kept decreasing over the day, with the lowest concentration at night. The enterohepatic circulation and glucuronidation process, which are regulated by the circadian clock may explain the differences in absorption of the compound depending on the time of administration.

## Obstacles and limitations

In spite of promising in vitro and in vivo results, certain hurdles need to be overcome before resveratrol can be widely considered in humans. It is important to emphasize that the dose used in the majority of in vitro studies, which is 100 µM [32, 37, 41, 43], is not physiologically relevant in humans. The systemic bioavailability of resveratrol is very low and administration of high doses is needed to reach a significant concentration in the blood [76] due to rapid metabolization in the intestine and the liver [77]. This leads to difficulties in transition to in vivo studies and shows that the promising impact on various diseases performed in in vitro studies must be treated with caution. In the context of disease prevention, resveratrol properties are often overestimated when it is taken only from natural sources, since the concentration in food and drinks is very low [78] and it is estimated not to exceed 50 nM [79]. The administration of the pure compound at high doses in the form of capsules did not improve significantly the bioavailability [76]. After oral administration of 150 mg of resveratrol to humans, the mean peak plasma concentration was of only 63.8 ng/mL [76]. Modifications using micronization [80], encapsulation or nanoparticles [81] have been used to overcome this hurdle. Micronized resveratrol was better absorbed when taken orally compared to the non-modified compound [80]. In another study, the administration of 5 g of micronized resveratrol resulted in a plasma concentration of 4.2 µM [82]. Another option could be to change the administration route, e.g. intravenous, intraperitoneal, or, less invasive, intranasal. Intranasal administration significantly improved the bioavailability of resveratrol in the lungs when given to mice compared to the oral route, and showed a significant decrease in tumor load in a lung cancer model [83]. The development of formulations that can be used in humans is also limited by resveratrol low solubility in water and other solvents. Besides bioavailability and solubility, another factor to consider is a time- and dose- dependent activity of resveratrol reported in some studies. One of them showed that the pro- and antioxidant properties of resveratrol were dependent on day or night administration [58]. The prooxidant activity of resveratrol was also shown in other studies, suggesting that the dose, cell type and presence of certain metal ions, such as copper, are important factors affecting the treatment outcome [84].

Given all of this, it is not surprising that only around 100 clinical trials were completed to date (https://www.clinicaltrials.gov/) and among them only one study alluded to chronobiology [76]. Resveratrol is currently available as a dietary supplement in many countries, however it is barely used in medicine. Therefore, future work should concentrate on its chronobiology, solubility and delivery to facilitate the transition to research in humans.

## Conclusions and perspectives

Most of the studies implied that resveratrol exerted a positive effect on circadian regulation and pathologies associated with circadian misalignment through SIRT1 up-regulation. Therefore, resveratrol may represent a nutraceutical useful in a wide variety of circadian-related disorders. Nonetheless, attention should be paid to the need for an improvement in bioavailability and delivery systems. This review will hopefully contribute to a greater awareness of the importance of circadian rhythm and chrononutrition in therapies but also in everyday life, as our lifestyle plays a pivotal role in clock disturbances.

## Data Availability

Not applicable.

## Abbreviations

BMAL1: brain and muscle Arnt-like protein 1
CAT: catalase
CLOCK: circadian locomotor output cycles kaput
CRY: cryptochrome
FFA: free fatty acids
GDF11: growth differentiation factor 11
GPR50: G protein coupled receptor 50
GRα: glucocorticoid receptor α
GPx: glutathione peroxidase
GST: glutathione S transferase
IRS-1: insulin receptor substrate 1
NQO1: NAD(P)H quinone dehydrogenase 1
Nrf2: nuclear factor erythroid 2-related factor 2
PER: period circadian protein
PGC-1α: peroxisome proliferator-activated receptor gamma-coactivator-1α
PP2A: protein phosphatase 2
REV-ERB: reverse erythroblastosis virus
ROR: retinoid-related orphan receptor
ROS: reactive oxygen species
SCN: suprachiasmatic nucleus
SIRT1: sirtuin 1
SOD: superoxide dismutase
